# Probabilistic modeling and analysis of the effects of extra-cellular matrix density on the sizes, shapes, and locations of integrin clusters in adherent cells

**DOI:** 10.1186/2046-1682-4-15

**Published:** 2011-08-09

**Authors:** Erik S Welf, Ulhas P Naik, Babatunde A Ogunnaike

**Affiliations:** 1 Department of Chemical Engineering, 150 Academy St, Colburn Lab, University of Delaware, Newark, Delaware 19716, USA; 2 Department of Biological Sciences, 309 Wolf Hall, University of Delaware, Newark, Delaware 19716, USA

## Abstract

**Background:**

Regulation of integrin binding to the specific complementary sites on extra-cellular matrix (ECM) proteins plays a major role in cell adhesion and migration. In addition to regulating single integrin-ligand bonds by affinity modulation, cells regulate their adhesiveness by forming integrin clusters. Although it is clear that cells exhibit different adhesion and migration behaviors on surfaces coated with different concentrations of ECM proteins, it is not clear if this response is mediated by changes in the availability of integrin binding sites or by differential intracellular signaling that may affect integrin binding and clustering.

**Results:**

To quantify how the concentration of ECM affects integrin clustering, we seeded cells expressing the integrin αIIbβ3 on different concentrations of the complementary ECM protein fibrinogen (Fg) and measured the resulting integrin cluster properties. We observed heterogeneity in the properties of integrin clusters, and to characterize this population heterogeneity we use a probabilistic modeling approach to quantify changes to the distributions of integrin cluster size, shape, and location.

**Conclusions:**

Our results indicate that in response to increasing ECM density cells form smaller integrin clusters that are less elongated and closer to the cell periphery. These results suggest that cells can sense the availability of ECM binding sites and consequently regulate integrin clustering as a function of ECM density.

## Background

Integrins are transmembrane adhesion receptors that facilitate cell adhesion by binding extra-cellular ligands to provide a mechanical linkage between a cell and the extracellular matrix (ECM). Many types of integrins form micron-sized clusters, which create the foundation for various cell-matrix adhesion complexes including focal adhesions. These complexes are populated by a diverse group of membrane, structural, adaptor, and enzymatic proteins [1], and signaling via these complexes affects many important cellular processes [2]. Integrin clusters thus provide the platform for signal propagation as well as force transduction through focal adhesions; as a result cell signaling and adhesion depend directly on the spatial and temporal characteristics of integrin cluster formation and dispersion [3-6].

Because integrin binding, clustering, and signaling depend on the availability of insoluble extracellular ligands [7-9], the availability of integrin binding sites is a critical property of the ECM proteins to which cells adhere. There is also a growing body of experimental evidence indicating that cells sense and respond to the concentration of ECM ligands available to them. Cell migration speed reaches a maximum at intermediate ECM density [10-13], and recent evidence suggests that the relationship between cell migration speed and ECM density may be mediated in part by a balance between integrin-mediated cell adhesion forces and myosin-mediated cell contractility [14]. The spacing between integrin ligands also affects cell spreading and migration [15], and grouping of integrin ligands in a clustered pattern has been shown to decrease the overall density of ligands necessary to support cell migration [16], suggesting that the local density of integrin ligands is more important than the global density. Cells also exhibit a phenomenon known as haptotaxis, or cell migration in response to a concentration gradient of adhesion ligand [17,18], a behavior that clearly requires the ability to direct cell migration in response to changes in ECM density.

Although it is clear that cells can sense and respond to different concentrations of ECM proteins adsorbed to a surface, it is unknown if this behavior is simply a result of differences in the number of integrin-ECM bonds and the resulting decrease in adhesion strength, or if cells can sense the availability of ECM binding sites and respond accordingly by regulating focal adhesion dynamics. Moreover, it is currently unknown what effect ECM density has on the clustering behavior of integrins. Given the important role of integrin clustering in supporting and regulating cell adhesion and migration [19-22], it is essential to understand how ECM density affects integrin clustering and ensuing focal adhesion formation. In this work, we characterize how integrin clustering changes as a function of ECM density by measuring the properties of integrin clusters formed in cells adhering to different concentrations of ECM protein. By implementing a labeling, measurement, and analysis technique designed specifically to identify bound integrins accurately, we are able to quantify the differences in integrin clusters present in cells adhering to different concentrations of ECM proteins.

Cluster properties such as size, shape, and location within the cell, are intrinsically non-uniform, showing significant variability *within *the same cell and *between *cells. Any attempt to characterize such heterogeneous population properties with their respective averages, while convenient, will largely be ineffective: large variability will obscure changes to mean cluster properties, making it difficult to determine with reasonable precision, the effects of different experimental variables on integrin clustering behavior. Proper characterization of the effect of different experimental conditions on integrin properties *cannot *be based on mean properties only; instead, we propose the use of appropriate probability distribution models to characterize the population behavior of integrin cluster size, shape, and location within the cell. The parameters of the probability distribution models used to describe integrin clusters in cells adherent to different concentrations of ECM are then used to quantify how cells alter integrin clustering behavior in response to adhesion on different concentrations of immobilized ECM proteins.

## Results

### Quantification of Integrin Cluster Properties

To investigate the effect of ECM density on integrin clustering, we used immunofluorescence microscopy to visualize integrin clusters in CHO cells adhering to coverslips coated in different concentrations of Fg. Integrin clusters were identified by labeling the β3 subunit of the integrin αIIbβ3, which the CHO cells were stably expressing. Although the αIIbβ3 heterodimer is not native to fibroblastic cell types such as CHO cells, this experimental system was chosen to restrict our analysis of integrin localization to a specific interaction between a single integrin and its ligand, thus enabling immunofluorescent labeling of all bound integrins with a single antibody. Because native CHO integrins cannot bind Fg, integrin-ECM interactions that result in integrin binding must be a result of αIIbβ3-Fg interactions. Additionally, the αIIbβ3 integrin has served as a model for integrin function for decades [17], and it has been shown that CHO cells expressing αIIbβ3 exhibit normal spreading and adhesion complex formation on Fg [18]. CHO cells stably expressing αIIbβ3 adhered and spread to Fg-coated coverslips, but not to those coated with BSA (results not shown), confirming that cell adhesion depends upon a specific interaction of αIIbβ3 with Fg.

**Figure 1:**
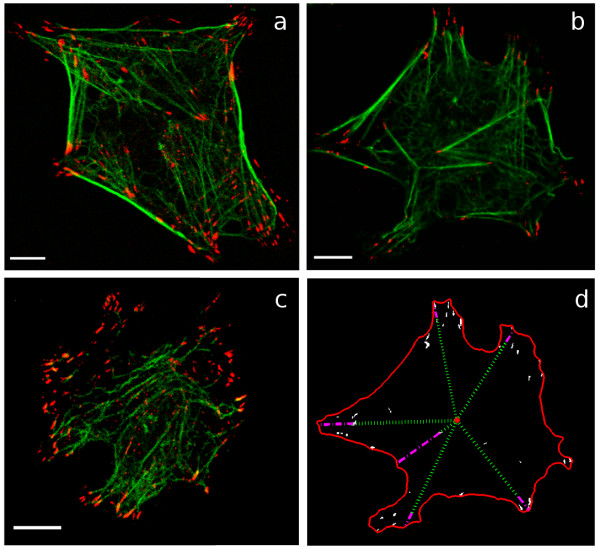
**Images of cells showing localization of integrins and heterogeneity in integrin cluster size, shape, and location**. Cells shown are plated on coverslips coated with: (a) 5 μg/mL Fg, (b) 20 μg/mL Fg, and (c) 100 μg/mL Fg. In (a), (b), and (c), integrin β3 is shown in red and F-actin is shown in green, and scale bars are 5 μm. (d) shows the cell from (b) following image analysis; white areas are integrin clusters, the red outline indicates the cell periphery, and the red circle identifies the cell centroid. The dotted green lines shows the distances from the cell centroid to several integrin clusters, and the dashed magenta lines show the corresponding distances from the integrin cluster to the cell periphery. The combination of the green line and the magenta line indicates the total distance from the cell centroid to the cell perimeter.

To visualize integrins that are bound to immobilized Fg, we implemented a crosslinking and extraction procedure described previously [23]. Comparison of the localization of integrins identified by the crosslinking and extraction procedure with integrins identified by conventional fixation and immunolabeling indicates that the crosslinking and extraction procedure does not substantially alter integrin localization [24]. The method used in this work was adapted from the method of Keselowski and Garcia [24] so as not to remove the entire cell cytoskeleton; this procedure resulted in images that clearly show small regions of high integrin concentration, predominantly located at the ends of cytoskeletal stress fibers. Remnants of the cell cytoskeleton were left so that the periphery of adherent cells could be identified easily during image analysis. Although an extraction method that removes more of the cell body and cytoskeleton may provide easier access for the antibody to bind to integrin cytoplasmic tails, our objective was not necessarily to identify all integrins in a focal adhesion, but rather to identify the borders of integrin-containing regions for the purpose of quantifying the cluster size, shape, and location accurately. The technique employed in this work effectively avoided non-specific staining and produced clear images without excessive staining of the cell body, as shown in Figure 1. Three example images of cells adhering to 5 μg/mL Fg, 20 μg/mL Fg, and 100 μg/mL Fg, are shown in Figure 1a, b and Figure 1c, respectively.

Automated analysis of images taken of cells adhering to 2-200 μg/mL Fg produced a data set describing integrin cluster properties in populations of cells adhering to different concentrations of Fg. Integrin clusters were identified based on standard image segmentation techniques as described in the Methods section; the size, shape, and location for each integrin cluster within each cell image were then calculated as described in the Methods section and as shown in Figure 1d.

Because cells were fixed before imaging, there was no way to determine what dynamic processes may have been affecting integrin clustering at the time of fixation. Any given integrin cluster may have been forming, maturing, or dispersing as a result of the individual cell's internal state or the local microenvironment at a given location within the cell. As a result, the population of integrin clusters in question is intrinsically heterogeneous because the processes that regulate integrin clustering are heterogeneous from cell to cell and even within the same cell. Indeed, visual inspection of the cell images and frequency histograms of the measured integrin cluster properties confirms this intrinsic heterogeneity in integrin cluster properties. Such heterogeneity obscures differences between cluster properties at different ECM concentrations by increasing the uncertainty associated with comparisons between population means under different conditions. Thus, given that the heterogeneity observed in integrin cluster measurements is intrinsic and is best characterized with probability distributions, not by averages alone, we employed probabilistic modeling and analysis techniques to describe integrin cluster properties. Changes in integrin cluster properties due to ECM density are then tracked with much better precision via the various probability distribution parameters.

### Integrin Cluster Size

**Figure 2:**
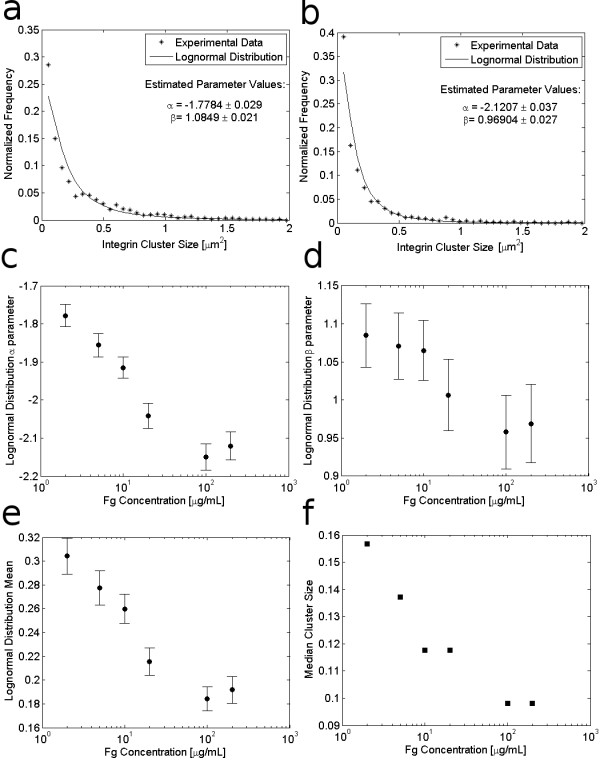
**Effect of Fg concentration on integrin cluster size**. The upper panels show example empirical frequency distributions for integrin cluster size in cells adhering to (a) 2 μg/mL Fg and (b) 200 μg/mL Fg along with the corresponding lognormal distributions. The lognormal distribution parameters, which were determined via maximum likelihood estimation from integrin cluster sizes in cells adhering to 2-200 μg/mL Fg, are shown in (c) and (d) along with 95% confidence intervals on the parameter estimates. (e) shows the calculated expected (mean) value for the distribution of integrin cluster sizes in cells adhering to each Fg concentration and (f) shows the median value of the integrin cluster sizes in cells adhering to each Fg concentration.

We postulated that integrin cluster size follows a lognormal distribution because size distributions arising from nucleation and growth processes typically follow the lognormal distribution [25-27] and it has been suggested that integrin clustering is a nucleation and growth process [3,28]. Additionally, the lognormal pdf has applications throughout the natural sciences and is often used to describe particle or droplet size distributions that arise as a product of a large number of independent, identically-distributed variables, such as particle breakage or growth [29]; thus, the lognormal distribution is a logical pdf to use for representing cluster sizes that are affected by a large number of protein-protein interactions, such as integrin cluster growth. Figures 2a and 2b show example empirical frequency distributions and corresponding lognormal distribution fits for integrin cluster sizes in cells adhering to 2 μg/mL Fg and 200 μg/mL Fg, respectively, confirming that integrin cluster sizes follow a lognormal distribution [23]. (Plotting these size distribution data on logarithmic axes facilitates visual comparison of the empirical data with postulated size distribution models. For example, Additional File 1 Figure S1 shows such a comparison of the lognormal distribution fit with that of the exponential distribution, an alternative model often postulated for size distributions. Note that in this specific case, the lognormal model provides a better fit to the data.)

In this work, the lognormal distribution parameters were determined by maximum likelihood estimation, which resulted in better model fits to the empirical data than least squares optimization. The lognormal distribution parameters α and β represent the scale and shape of the distribution, respectively, and Figures 2c and 2d show how the estimated lognormal distribution parameters change with ECM density. The scale parameter α decreases significantly with increasing ECM concentration, indicating that the distribution scales down toward smaller sizes. Decreases in the shape parameter β with increasing ECM concentration also indicate a shift in the size distribution towards smaller, but more symmetrically distributed clusters [30]. Overall, the changes in the fitted lognormal distributions indicate that with increasing ECM concentration the frequency of occurrence of integrin cluster sizes larger than 0.2 μm^2 ^decreases while the frequency of clusters smaller than 0.2 μm^2 ^increases. Figure 2e shows that the calculated expected, or mean, value of the lognormal distribution, given by , decreases with increasing ECM concentration. For lognormally-distributed random variables the population median provides a better indication of the central location of the population than the population mean [30], and Figure 2f shows that the median cluster size also decreases with increasing ECM density. The observation that integrin cluster size decreases with increasing ECM density is in agreement with a previous study showing that the size of paxillin-containing adhesions in PtK_1 _cells decreased in response to increases in fibronectin coating density [14], but to our knowledge this is the first result showing changes in the size of adhesion structures based on integrin labeling.

### Integrin Cluster Location

**Figure 3:**
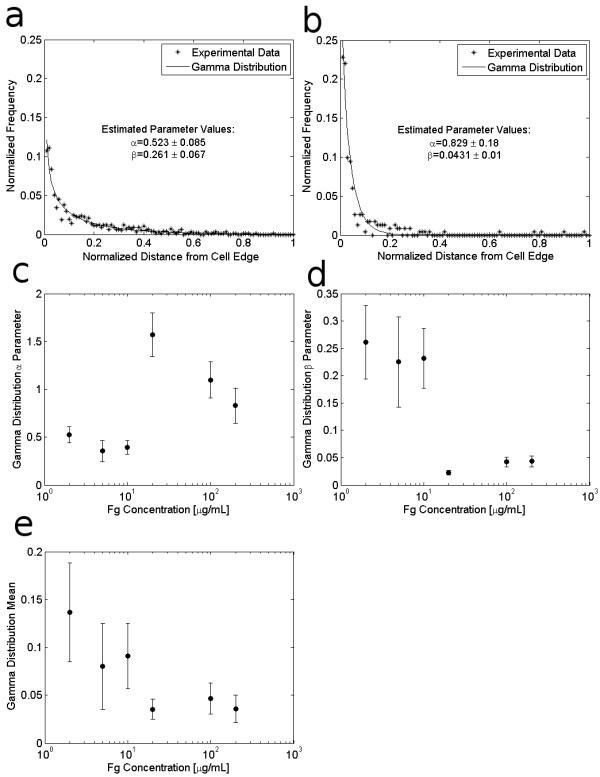
**Effect of Fg concentration on integrin cluster distance from cell edge**. Integrin cluster location is measured as the distance of each cluster from the cell edge, normalized by the distance from the cell center to the cell edge. Two example frequency distributions and estimated gamma probability distribution functions for integrin clusters > 0.5 μm^2 ^are shown for cells adhering to: (a) 2 μg/mL Fg, and (b) 200 μg/mL Fg. The gamma distribution parameters α and β which were determined by least squares fitting of the gamma distributions to the frequency distributions of locations of integrin clusters in cells adhering to 2-200 μg/mL Fg, are shown in (c) and (d) along with 95% confidence intervals on the parameter estimates. (e) shows the calculated expected (mean) value for the distribution of integrin cluster location in cells adhering to each Fg concentration.

**Figure 4:**
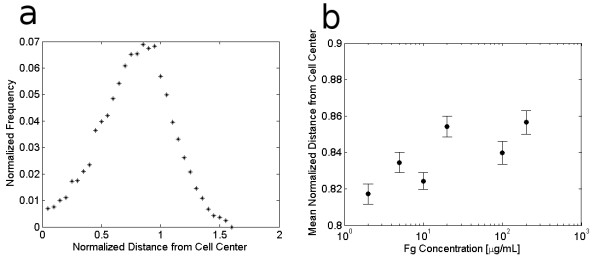
**Effect of Fg concentration on integrin cluster distance from cell center**. The distance of each integrin cluster from the cell centroid is normalized by the average radius of that cell, which is defined as the mean of the distances from the cell centroid to the cell perimeter, as measured at each cluster location (see Figure 1d). Based on this normalization, most integrin clusters are located just inside the cell periphery at a normalized cluster location below 1; however, some clusters may reside at a normalized cluster location greater than 1 if they are located further from the cell centroid than the average distance to the cell periphery. (a) shows an example frequency distribution of normalized cluster locations in cells adhering to 2 μg/mL Fg. (b) shows that integrin cluster locations move closer to the cell periphery as ECM density increases.

Because the specific positions where integrin clusters are located within a cell determine the cell's ability to transfer force to the ECM and support cell spreading and migration, this particular integrin cluster characteristic provides insight into how a cell distributes the forces necessary for cell adhesion and migration into discrete contact points that populate the cell-matrix interface [31]. To quantify integrin cluster location, we measured the distance of each integrin cluster from the cell edge, normalized by the length of the line from the cell centroid to the cell edge and passing through the cluster as illustrated in Figure 1. As noted in [30] the waiting times between randomly occurring (i.e. Poisson distributed) events tend to follow a gamma distribution. Thus if we equate waiting time between cluster formation events to distance moved by the cell edge after nucleation of an integrin cluster at the cell's leading edge, we expect the distances between integrin clusters and the cell edge to follow a gamma distribution. Indeed, as shown in Figures 3a and 3b, the frequency distribution of these measured radial distances appears to follow a gamma distribution. The indicated parameters, determined by least squares fit to the empirical distribution, provided a better match to the data than parameters estimated via maximum likelihood. Although no clear trend in the gamma distribution parameters was evident when the gamma pdf was fit to the locations of integrin clusters of all sizes (data not shown), when the analysis is restricted to cluster sizes larger than 0.5 μm^2^, the estimated gamma distribution parameters showed distinct trends with increasing ECM density, as shown in Figures 3c and 3d. From a fundamental relationship between gamma-distributed and Poisson-distributed random variables, we are able to draw the following insight: the parameter α of the gamma distribution model for integrin cluster location represents the total number of Poisson-distributed events that have occurred in forming the cluster, with the parameter β as the inverse of the mean rate of occurrence of these events [23,30]. The trends shown in Figures 3c and 3d suggest that the rate and number of events leading to cluster formation increase at higher ECM density, suggesting that such events might be related to the availability of ECM binding sites. Figure 3e shows that the expected value of the gamma distribution, αβ, decreases with increasing ECM density, indicating that integrin clusters are closer to the cell periphery in cells adhering to higher ECM density. We also quantified integrin cluster location by measuring the cluster distance from the cell centroid, normalized by the average radius of the cell. As shown in Figure 4, the mean value of this metric increases with increasing ECM density, confirming that integrin clusters are closer to the cell edge in cells adhering to higher ECM concentrations.

### Integrin Cluster Shape

Because integrin clusters have been shown to grow anisotropically as a function of an applied force, the cluster shape can provide a valuable measure of local stresses within a cell [32,33]. We quantify the shape of an integrin cluster with "eccentricity" as determined by the Matlab function *regionprops*. The cluster is idealized as an ellipse having the same normalized second central moment as the coordinates of the pixels within the integrin cluster, and the eccentricity of this "equivalent ellipse" is determined, by definition, as the ratio of the distance between the foci of the ellipse and its major axis length. By the definition of eccentricity, a value close to one indicates an elongated cluster; a value close to zero indicates a circular cluster. In our analysis, integrin clusters less than 0.1 μm^2 ^in size are identified by fewer than 5 contiguous pixels and as a result clusters smaller than 0.1 μm^2 ^can only exhibit one of a few discrete values for eccentricity. To prevent this data quantization from skewing the cluster eccentricity distribution towards any of the discrete eccentricity values exhibited by small clusters, integrin clusters smaller than 0.1 μm^2 ^are excluded from our analysis of integrin cluster shape.

**Figure 5:**
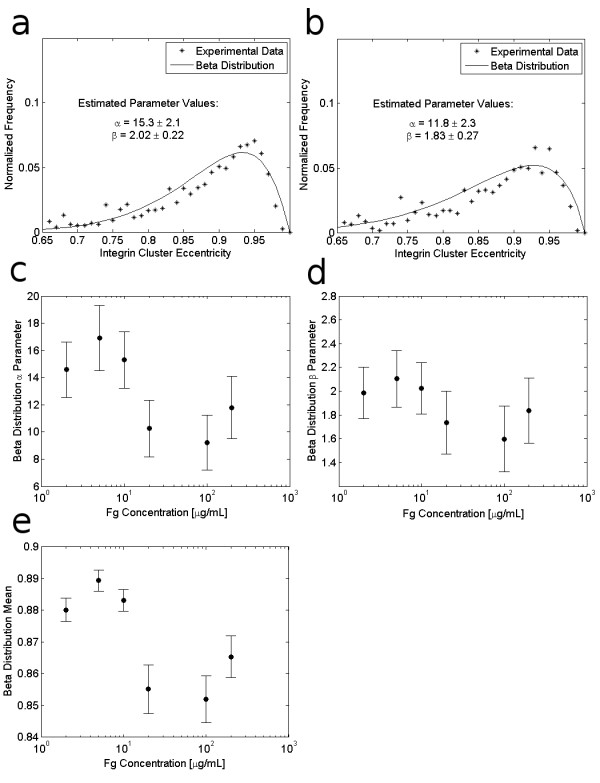
**Effect of Fg concentration on integrin cluster shape**. Integrin cluster shape is characterized by the cluster eccentricity. (a) and (b) show example empirical frequency distributions and corresponding beta distributions for integrin clusters in cells adhering to 10 μg/mL Fg, and 200 μg/mL Fg, respectively. (c) and (d) show the effect of ECM density on the least squares estimates for the beta distribution parameters, along with 95% confidence intervals for the parameter estimates. (e) shows the calculated expected (mean) value for the distribution of integrin cluster shapes in cells adhering to each Fg concentration.

The fact that eccentricity is constrained by definition to lie between 0 and 1 suggests that the distribution of integrin cluster eccentricities will be well-characterized by the beta distribution, a distribution that is optimal for characterizing random variables that are scaled between 0 and 1 [23,30]. Thus, in the absence of additional information about explicit biophysical mechanisms driving the distribution of integrin cluster shapes, we employ the beta distribution to quantify integrin cluster eccentricities. Figures 5a and 5b show example empirical frequency distributions for cluster eccentricity along with beta distribution fits for clusters in cells adhering to 10 μg/mL and 200 μg/mL of Fg, respectively. Classically, focal adhesions and integrin clusters have been described as elongated adhesion structures, and the illustrative distributions in Figures 5a and 5b confirm that most integrin clusters have an elongated shape, with eccentricity close to 1. As shown in Figures 5a and 5b, cells adherent to higher concentrations of Fg also exhibit increased heterogeneity in cluster shape. Figures 5c and 5d show that estimates of the beta distribution parameters decrease slightly with increasing Fg density, and Figure 5e shows that the expected value for the beta distribution, given by , decreases with increasing ECM density, indicating that integrin clusters are generally more elongated at lower ECM concentrations. (The beta distribution parameters shown were estimated via least squares; they provided better fits to the empirical data than parameters obtained via maximum likelihood estimation.)

**Figure 6:**
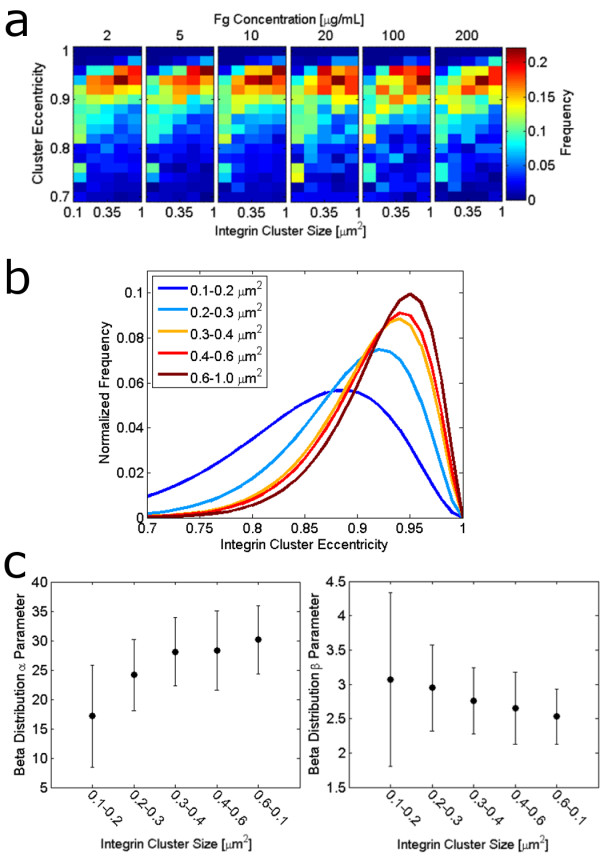
**Relationship between cluster size and distributions of cluster shapes**. (a) shows a heatmap of the normalized empirical frequency distribution for integrin cluster shape, characterized by eccentricity, for different ranges of integrin cluster sizes. The cluster sizes separated into columns in all panes of (a) are: 0.1-0.2 μm^2^, 0.2-0.3 μm^2^, 0.3-0.4 μm^2^, 0.4-0.6 μm^2^, 0.6-1 μm^2^. (b) shows the fitted beta probability distributions for integrin clusters of different sizes in cells adhering to 10 μg/mL Fg, and (c) shows the corresponding estimated beta distribution parameters along with 95% confidence intervals on the parameter estimates.

Integrin clusters grow in response to contractile forces transferred to adhesions by the cell cytoskeleton, causing focal adhesions to grow anisotropically in the direction of applied force [34,35]. Recently, it has become clear that nascent adhesions, which typically form in the actin-rich lamellipodium, experience different forces when compared with larger, more elongated clusters that are commonly associated with actin stress fibers in the cell lamella [36,37]. Presumably, the adhesions that are subject to the greatest contractile force during the initial stages of adhesion maturation become the largest and most elongated, and they maintain their size even after decreases in local stresses [38]. In contrast, the small adhesions that have not experienced such contractile forces retain the symmetry typical of small nascent adhesions [37]. This differential application of force to integrin-based adhesions may be a cause for heterogeneity in integrin cluster shapes; thus it was of interest to determine if clusters of different sizes exhibit different shapes. Figure 6a shows a heatmap of the empirical frequency distributions of integrin clusters separated according to different size ranges. The observation that there is a higher frequency of clusters with eccentricity between 0.92 and 0.94 and a lower frequency of clusters with eccentricity between 0.84 and 0.86 at larger integrin cluster sizes suggests that larger clusters tend to be more elongated. Indeed, comparison of the beta pdfs fit to the eccentricity frequency distributions of different size ranges of integrin clusters, as shown in Figure 6b, clearly shows that larger integrin clusters have higher eccentricities than smaller clusters. The estimated beta distribution parameters corresponding to the distributions shown in Figure 6b are shown in Figure 6c.

## Discussion

Focal adhesions and integrin clusters are dynamic structures that change in response to a variety of biochemical and mechanical cues. However, because the properties of these adhesion structures are difficult to quantify, systematic analysis of integrin cluster properties has been difficult. Given the population heterogeneity observed in the integrin cluster properties of adherent cells, the characteristics of the entire population of integrin clusters in adherent cells must be used if the effects of different treatment conditions on integrin clustering are to be determined properly. Further, appropriate probability models must be used to quantify changes to the population as a result of treatment conditions. In this study, we have used a combination of specialized protein labeling techniques, custom image analysis programs, and probabilistic modeling, to show how population heterogeneity may be included in overall measures of heterogeneous cellular structures. It is expected that such modeling approaches will prove useful in analyzing other cellular systems where population heterogeneity obscures changes to ensemble statistics used to quantify heterogeneous properties. For example, the data shown here represent only a single time point taken during the dynamic processes of cell adhesion, spreading, and migration; studying how the distributed properties of integrin clusters change during the dynamic processes of spreading and migration should provide valuable insight into how adhesions structures may be remodeled differentially in response to different variables such as ECM density, substrate stiffness, or soluble factors.

Here we argue that if integrin clustering is a nucleation and growth process, integrin cluster size would be represented well by a lognormal distribution, and our experimental data support this argument. Alternatively, it has been suggested that the sizes of focal adhesions that are subject to only anisotropic growth may follow an exponential distribution [39]; however because our data set includes small integrin clusters that are presumably not subject to anisotropic growth we do not observe an exponential distribution of integrin cluster size (Additional File 1 Figure S1). Our sample population was designed to include integrin clusters of all sizes; as a result, only a small fraction were of the large elongated variety that are likely subject to anisotropic growth and are predicted to follow an exponential size distribution [39]. Because of the low number of larger integrin clusters, we were unable to determine the size distribution for just the large clusters. To test the hypothesis that large integrin clusters follow an exponential size distribution, experimental conditions will need to be altered to obtain more large clusters or a much larger sample would be required to determine the size distribution of large clusters.

Although it is somewhat counterintuitive that cells form smaller, less elongated integrin clusters when adhering to higher concentrations of ECM, this observation has important implications for inferring the mechanisms responsible for cellular regulation of integrin clustering. Because one would expect an increase in the number of binding sites to increase the extent of integrin binding, the observation that integrin cluster size decreases with increasing ECM density suggests that cells internally regulate integrin cluster size in response to changes in ECM density. Indeed, our analysis suggests that cells exhibit more total area occupied by bound integrins when adhering to surfaces coated with lower ECM than on surfaces coated with higher ECM (Additional File 2 Figure S2). It is tempting to propose that the increase in integrin cluster size at lower ECM densities is due to a decrease in the number of clusters; however, we have been unable to identify any clear trend in the number of integrin clusters per cell in response to increasing ECM density (data not shown). An alternative explanation for the observed results is that cells may form larger integrin clusters when there are fewer integrin binding sites as a result of differential outside-in signaling from integrins [40] or as a result of decreased intracellular mechanotransduction caused by differences in ECM rigidity at higher ECM density [41-43]. Additional experimental observations also support the alternative explanation that some intracellular component is limiting during adhesion [44]. Although increases in adhesive area increase adhesion strength when the starting area is low, cell adhesion strength exhibits saturation with respect to increasing adhesive area [44]. Because the number of integrin clusters does not seem to be related to cluster size, it is reasonable to assume that the availability of unbound integrins is not a limiting factor in integrin cluster growth, and our observations provide further evidence that integrin cluster growth is not limited by binding site availability at the Fg concentrations we evaluated. Thus the conclusion that integrin clustering is not limited by the availability of integrins or integrin binding sites suggests that cells internally regulate integrin clustering by some other means.

## Conclusions

The results presented in this paper collectively suggest that cells respond to the availability of ECM binding sites by regulating integrin cluster properties, either as a function of the stress that is applied to each integrin cluster or as a result of differential intracellular signaling. If cellular processes governing integrin cluster formation, growth, or turnover are regulated by integrin outside-in signaling that occurs in response to the extent of integrin ligation, then such mechanisms could be the means by which cells regulate integrin clustering. Regulation of focal adhesion size by the integrin-associated protein focal adhesion kinase (FAK) is an example of how integrin-mediated signaling may regulate focal adhesion size [45]. The frequency of interaction between cells and the ECM is an attractive mechanism by which cells can sense the availability of ECM binding sites; if this interaction results in outside-in signaling events that are integrated by the cell, then the cell may use this information to regulate integrin clustering in response to the ECM density. Because of their outside-in signaling capacity, integrins function not only as regulators of cell adhesion but also as sensors of their extracellular environment. What we have provided here is evidence suggesting that cells do just that: sense the concentration of ECM on a surface and regulate their adhesion structures in response to this information.

## Methods

### Experimental

To study the spatial distribution of integrins, we utilized a technique that labels only integrins bound to immobilized ECM protein and minimizes non-specific staining of the cell body [24]. Chinese Hamster Ovary (CHO) cells were transfected using Lipofectamine (Invitrogen) following the manufacturer's protocol. Geneticin (G418) was added to the culture medium 24 hours after transfection at a final concentration of 500 μg/mL. The resistant colonies were isolated to obtain single cell clones, and cells stably expressing the integrin αIIbβ3 were maintained in Dulbecco's Modified Eagle Medium (DMEM) containing 10% Fetal Bovine Serum (FBS) and 300 μg/mL G418. Cells were serum starved with DMEM containing 0.5% Bovine Serum Albumin (BSA) 12 hours prior to treatment. Glass coverslips were prepared for the cell adhesion assay by treating with various concentrations of fibrinogen in Phosphate Buffered Saline (PBS) for 12 hours at 4°C prior to cell adhesion. Serum starved cells were released from culture dishes with Versene and washed once with 0.5% BSA in DMEM. Cells were then plated onto coverslips coated with Fg and incubated at 37°C for 2 hours. Coverslips were then washed once with PBS, and extracellular proteins were crosslinked with 0.4 mM bissulfosuccinimidyl suberate (BS3), a cell-impermeable crosslinker, for 15 minutes. The crosslinking reaction was quenched with 10 mM Tris for 2 minutes and washed twice with PBS. Uncrosslinked proteins were then extracted with 0.5% NP-40 in PBS for 10 minutes, and then coverslips were washed twice with PBS. The remaining proteins were then fixed using 4% paraformaldehyde for 10 minutes, and washed twice with PBS. Coverslips were blocked overnight at 4°C using 3% bovine serum albumin (BSA) in PBS, then incubated with primary antibody, goat anti-integrin β3 IgG (C-20) from Santa Cruz, for one hour at 37°C. Coverslips were then washed 3 times with PBS and incubated with secondary antibody, Alexa Fluor 568-conjugated donkey anti-goat IgG from Invitrogen, along with fluorescein-labeled phalloidin for one hour at 37°C, then washed an additional three times before mounting and imaging. Cell images were collected using a Zeiss confocal microscope with 63× oil objective. In order to avoid sampling bias during cell imaging, cells were selected at random for imaging without regard to perceived differences in integrin cluster characteristics. The only requirement for inclusion in the sample set was that cells exhibit a spread morphology.

Using the experimental protocol described above, we performed three replicates of the adhesion assay for each Fg concentration. Each replicate experiment was performed on a different day, using a different sample of cells from the same genetically identical population. Images of at least ten different cells were taken on each day for each experimental condition, resulting in a total of at least 35 cells and at least 5,000 integrin clusters measured per Fg concentration.

### Image analysis

All image processing and analysis was performed on the Matlab platform (Mathworks, Natick, MA) using custom-written image analysis code as described below. Only non-overlapping cells were analyzed, and images were cropped so that a single image represented a single cell of interest. Following image capture, each Zeiss image file was converted to an 8-bit Red-Green-Blue (RGB) tiff file, with each color channel representing either β3 integrin or the actin cytoskeleton.

Integrin clusters were identified by segmenting integrin intensity images as follows. Image pixels having intensity values above a manually-selected intensity threshold were segmented into a binary image. (The appropriate intensity threshold, which varied for different images and different cells, was selected manually for each cell image as described previously [23].) Integrin clusters were identified as groups of connected pixels in the segmented binary image. The properties of the regions identified by this method were then quantified using the Matlab image analysis function *regionprops*. Regions smaller than 0.02 μm^2 ^were excluded from our analysis because they represent regions consisting of fewer than two contiguous pixels.

To identify the cell body and cell periphery, images showing the intensity of both β3 integrins and the cell cytoskeleton were manually segmented by identifying an appropriate intensity threshold for each image. Images were then subjected to image dilation and subsequent image erosion to eliminate open spaces in the interior of the cell. This operation created a filled region representative of the entire cell body which was used to identify the cell centroid location, cell area, and cell edge via the Matlab functions *regionprops *and *bwperim*.

### Probability models

As shown in the cell images in Figure 1, integrin cluster properties are heterogeneous across different cells and even within the same cell. To describe the entire population of cluster properties fully, appropriate probability models were developed for integrin cluster size, shape, and location, as described previously [23]. Specifically, following arguments presented in [23], the distribution of integrin cluster area was represented with the lognormal probability distribution function (pdf), given by:(1)

We idealize integrin cluster shape as an ellipse and use its eccentricity, the ratio of the distance between foci of an ellipse and its major axes length, as a measure of cluster shape. The distribution of cluster eccentricity is then described by the beta pdf:(2)

where Γ(.) is the gamma function. The distribution of integrin cluster distance from the cell edge to the integrin cluster is represented with the gamma pdf:(3)

Recall that for each pdf shown above,  is the probability of the random variable *x *(cluster size, cluster eccentricity, or cluster location) taking values that lie between *a *and *b*. Each probability distribution function (pdf) has two model parameters, α and β, which were determined by fitting the models to our empirical data.

## Authors' contributions

ESW, UPN, and BAO designed the research and wrote the manuscript; ESW performed the experiments and analysis; all authors read and approved the final manuscript.

## Supplementary Material

Additional file 1 **Figure S1 - Comparison of empirical size distribution data with size distribution models**. Both panels show empirical frequency distributions for integrin cluster size in cells adhering to 5 μg/mL Fg. (a) shows the fitted lognormal distribution and corresponding parameters along with their 95% confidence intervals determined via maximum likelihood estimation, which in this case provides a better fit to the data than least squares estimation. (b) shows the fitted exponential distribution and corresponding parameter along with its 95% confidence interval determined via least squares estimation, which in this case provides a better fit to the data than maximum likelihood estimation. Click here for file

Additional file 2 **Figure S2 - Cell area occupied by bound integrins**. The total area occupied by bound integrins in cells adhering to different concentrations of ECM is shown, normalized by the number of cells analysed at Fg concentration. Click here for file
