# Supplementary figures and images for: Probabilistic modeling and analysis of the effects of extra-cellular matrix density on the sizes, shapes, and locations of integrin clusters in adherent cells
Source: BMC Biophys. 2011 Aug 9;4:15. doi: 10.1186/2046-1682-4-15 (PMC3179437; doi:10.1186/2046-1682-4-15)

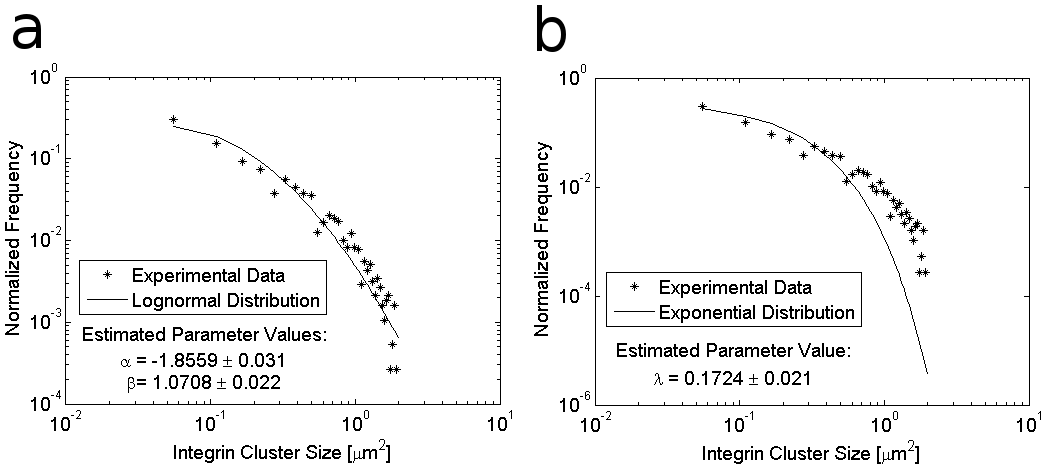

Supplement: Additional file 1 — Figure S1 - Comparison of empirical size distribution data with size distribution models. Both panels show empirical frequency distributions for integrin cluster size in cells adhering to 5 μg/mL Fg. (a) shows the fitted lognormal distribution and corresponding parameters along with their 95% confidence intervals determined via maximum likelihood estimation, which in this case provides a better fit to the data than least squares estimation. (b) shows the fitted exponential distribution and corresponding parameter along with its 95% confidence interval determined via least squares estimation, which in this case provides a better fit to the data than maximum likelihood estimation. [file 2046-1682-4-15-S1.TIFF]

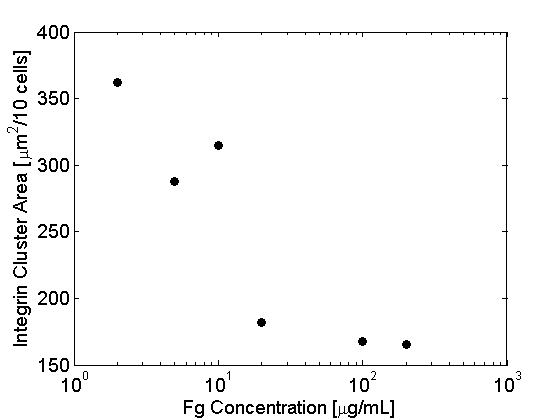

Supplement: Additional file 2 — Figure S2 - Cell area occupied by bound integrins. The total area occupied by bound integrins in cells adhering to different concentrations of ECM is shown, normalized by the number of cells analysed at Fg concentration. [file 2046-1682-4-15-S2.TIFF]
